# Changing demographics of visceral leishmaniasis in northeast Brazil: Lessons for the future

**DOI:** 10.1371/journal.pntd.0006164

**Published:** 2018-03-06

**Authors:** Iraci Duarte Lima, Adila L. M. Lima, Carolina de Oliveira Mendes-Aguiar, José F. V. Coutinho, Mary E. Wilson, Richard D. Pearson, José Wilton Queiroz, Selma M. B. Jeronimo

**Affiliations:** 1 Health Graduate Program, Health Science Center; Federal University of Rio Grande do Norte, Natal, RN, Brazil; 2 Institute of Tropical Medicine of Rio Grande do Norte, Federal University of Rio Grande do Norte; Natal, RN, Brazil; 3 State of Rio Grande do Norte Health Secretariat; Natal, RN, Brazil; 4 Center for Zoonosis Control, Natal Health Secretariat, Natal, RN, Brazil; 5 Departments of Internal Medicine, Microbiology and Epidemiology, University of Iowa, and the Veterans’ Affairs Medical Center, Iowa City, IA, United States of America; 6 Division of Infectious Diseases and International Health, Department of Medicine, University of Virginia, Charlottesville, VA, United States of America; 7 Institute of Science and Technology of Tropical Diseases, INCT-DT, Salvador, Brazil; 8 Department of Biochemistry, Bioscience Center, Federal University of Rio Grande do Norte, Natal, RN, Brazil; University of Queensland, AUSTRALIA

## Abstract

**Background:**

Visceral leishmaniasis (VL) caused by *Leishmania infantum* became a disease of urban areas in Brazil in the last 30 years and there has been an increase in asymptomatic *L*. *infantum* infection with these areas.

**Methodology/Principal findings:**

A retrospective study of human VL was performed in the state of Rio Grande do Norte, Brazil, for the period of 1990–2014. The data were divided into five-time periods. For all VL cases, data on sex, age, nutritional status and childhood vaccination were collected. Geographic information system tools and statistical models were used to analyze the dispersion of human VL. The mean annual incidence of VL was 4.6 cases/100,000 inhabitants, with total 3,252 cases reported. The lethality rate was 6.4%. Over time the annual incidence of VL decreased in the 0–4 years (*p*<0.0001) and 5–9 (p <0.0001) age groups, but increased in ages 20–39 (p<0.001) and >40 years (p<0.0001). VL occurred more often in males (β_2_ = 2.5; p<0.0001). The decreased incidence of VL in children was associated with improved nutritional status and childhood immunizations including measles, poliomyelitis, BCG, and hepatitis B. Human VL correlated temporally and geographically with canine *L*. *infantum* infection (p = 0.002, R^2^ = 0.438), with rainfall and with *Lutzomyia longipalpis* density (r = 0.762). Overall, the incidence of VL decreased, while VL-AIDS increased, especially between 2010–2014. VL was more frequently found in areas that lacked urban infrastructure, detected by lack of garbage collection and sewers, whereas HIV infection was associated with higher levels of schooling and evidence of higher socioeconomic status.

**Conclusion/Significance:**

The demographics of VL in northeastern Brazil have changed. Disease incidence has decreased in children and increased in adults. They were associated with improvements in nutrition, socioeconomic status and immunization rates. Concurrent VL-AIDS poses a serious challenge for the future.

## Author summary

We studied factors associated with the changing demographics of visceral leishmaniasis (VL) in Northeast Brazil, including environmental and socioeconomic determinants of disease, during the period 1990 to 2014. The incidence of VL was higher in urban areas, and regions with higher levels of canine *L*. *infantum* infection. Human males were more commonly affected than females. The incidence of VL in children under age 10 decreased during the period of study, simultaneous with increased incidence in adults. Reduction in the VL incidence among children was associated with improved socioeconomic status, administration of childhood vaccines and better nutritional status. Geographic areas with higher rainfall had higher densities of *Lu*. *longipalpis*, the primary vector of *L*. *infantum* in Brazil. VL was more frequently found in areas with indicators of poverty including sparse garbage collection and lack of urban infrastructure. During the past 25 years, HIV/AIDS has spread to areas where VL is endemic and has contributed to an increased incidence of VL-AIDS co-infection in adults.

## Introduction

Visceral leishmaniasis (VL) is a life-threatening disease caused by *L*. *infantum* [1], which was first recognized in Brazil in 1932 [2–4]. It is likely the parasite initially arrived in northeastern Brazil with people and/or dogs previously infected with *L*. *infantum* in southern Europe or North Africa [5;6]. *Lu*. *longipalpis* is a competent vector for *L*. *infantum*, and it is found in most countries in Latin America. Dogs are considered the major reservoirs of *L*. *infantum*. At first, cases of VL in Brazil occurred sporadically in semi-arid, rural areas of the northeast region. Most cases occurred in children under 10 years of age [7;8]. However, in the late 1980’s and early 1990’s, urban outbreaks occurred in large cities in the northeast and other regions of Brazil [9–12]. Massive migration of the population to urban regions, adaptation of *Lu*. *longipalpis* to peridomestic environments, and transport of *L*. *infantum* infected dogs to urban areas occurred during this period. The temporal occurrence of human VL has assumed a variable pattern that correlates with environmental forces including el Niño/la Niña [13;14], which influence rainfall and humidity and thus the density of sand flies.

Several studies in endemic areas of Brazil have shown that most people infected with *L*. *infantum* remain asymptomatic, if they are not immunosuppressed [15–19]. It is not clear whether people with VL or with asymptomatic *L*. *infantum* infection serve as reservoirs and contribute to the long-term maintenance of this pathogen in endemic areas. In the past, the ratio of symptomatic VL to asymptomatic *L*. *infantum* infection among children was approximately one in every six, and for adults the ratio was 1 in 18. Risk factors for developing symptomatic VL in children included malnutrition [20–22], neoplastic disorders [19;23] and viral co-infection [24]. Brazil has had freely available vaccination for all age groups since early 1980 and this has had a coverage of over 90% for routine child immunizations. This improved vaccination rate has led to decreased and/or elimination of some of the common and potentially fatal diseases of children. For instance, it is known that measles infection predisposes to opportunistic infection for years [25;26] and measles has been eliminated from Brazil for almost a decade, although there was an outbreak in 2013, which was contained [27;28].

HIV/AIDS is known to increase the risk of developing symptomatic, rather than asymptomatic VL [29–31]. HIV co-infection with VL was first noted to contribute to the increased incidence of VL in adults in Europe in the mid-1980’s [32]. VL/AIDS was recognized in Brazil in early 90’s [32–34]. Thus, a new epidemiological pattern of VL is emerging because of spread of HIV to all regions of the country. Subjects with VL-AIDS have an increased risk of VL relapse and death. The goal of the current study was to identify demographic, spatial and socioeconomic factors associated with VL in northeastern Brazil between 1990 and 2014, using VL cases reported in the state of Rio Grande do Norte. We assessed the geographic distribution of VL and its association with the spatial distribution of HIV/AIDS, and with socioeconomic factors that could influence the outcome of *L*. *infantum* infection. A better understanding of the epidemiological dynamics of *L*. *infantum* and HIV co-infections, and their determinants is essential to guide new health policies.

## Methods

### Study area

The study was conducted in the state of Rio Grande do Norte in northeastern Brazil. The state has an area of 52,811,126 square kilometers, with a population of 3,408,510, 77.8% of whom now live in urban areas. Most of the state has a semi-arid climate, with rainfall less than 800 mm per year and an average temperature of 27°C. A more humid climate is found along the east coast of the state, which borders the Atlantic Ocean, where the rainfall indices are greater than 1,400 mm per year. The state is grouped in 19 micro-regions (MR), each with distinct climate, topography, hydrography, population density and economy. These micro regions served as units for the current analysis.

### Study design

A retrospective study of VL cases diagnosed in the state of Rio Grande do Norte was performed, and correlated with demographic and epidemiologic factors in the corresponding regions of the state. The study was divided into five time periods: (1) 1990–1994, (2) 1995–1999, (3) 2000–2004, (4) 2005–2009 and (5) 2010–2014. The association between spatial patterns of VL and HIV-AIDS was assessed using quantitative data available from 1991, 2000 and the 2010 censuses. The incidence of VL, AIDS and VL-AIDS co-infection per 100,000 inhabitants was extracted from the state records listed below. Additional variables that were collected included: (1) dates of new cases of VL, AIDS and VL-/AIDS, (2) sex, (3) age, (4) nutritional status of children under 5 years, (5) vaccination rates for measles, poliomyelitis, BCG and hepatitis B in children under 5 years. The spatial and temporal correlation between human VL and canine infection was also assessed. Environmental or socioeconomic variables considered were: (1) the association of annual rainfall and the density of *Lu*. *longipalpis*, (2) socioeconomic data from the censuses included literacy rate, education, income, city water supply, waste disposal, septic tank and presence of sewage.

### Data collection

Data on human VL were obtained from the Notifiable Diseases Information System (SINAN). This federal government system catalogs the reported cases and coordinates investigations of diseases for which reporting is mandated by the Brazilian government, as defined by specific legislation. The data are captured in the Health Post Centers and/or hospitals and are sent to state Secretary of Health, whose office uploads the information in SINAN. The list of notifiable disease is updated as new outbreaks occur. For example, the recent epidemic of Zika virus infections led this virus to be added to the list of mandatory reportable diseases. Data on *Lu*. *longipalpis* density and rates of infection in domestic dogs were obtained from the Surveillance and Leishmaniasis Control Program, Secretary of Health. Data on HIV or AIDS were obtained by crossing information from SINAN with the Brazilian Mortality Information System database and release of medications for HIV. Nutritional data on children younger than 5 years were obtained from the Brazilian Minister of Health. Those data were available for all 19 micro-regions of the state of Rio Grande do Norte.

Data about immunization coverage were obtained from the Brazilian National Program of Immunization Information System. The vaccination coverage percentage was calculated per estimated population at the age group targeted to be vaccinated and the doses of vaccines administered in each municipality, and grouped into the 19 micro regions for this analysis.

Data variables gathered from censuses included education level, income, local health facility, piped water supply, garbage collection, street cleaning, sewer system, septic tank, urbanization and population density. Those data were collected from the Instituto Brasileiro de Geografia e Estatística (IBGE) website (http://www.ibge.gov.br/home/). Annual rainfall data in the municipalities were obtained from the State of Rio Grande do Norte Agricultural Research Company, EMPARN, (http://www.emparn.rn.gov.br/).

### Statistical analysis

The effect of sex and micro-region (**MR**) on the temporal variation in the incidence VL from 1990 to 2014 were evaluated by a general linear model with categorical explanatory variables [35] according to the following formula: *Y_ti_* = *β*_0_ + *β*_1_*t* + *β*_2_*I*(*Sex*) + *θ_i_****MR***(*i*|19) + *error*, (*model* 1) where the dependent variable was the incidence of VL (*Y*_*ti*_) per 100,000 inhabitants, in the year t and in the micro-region i. The independent variables were the time (t) in years, the categorical variables were sex *(1 if male)* and micro-region (1–19) **MR** (*i |19)*. The β1 coefficient measured the average annual increase in the incidence of VL, whereas β2 measured the differential incidence of VL between male and female. Micro-region 19 was considered the reference, since it was the site of the first VL outbreak in the state of Rio Grande do Norte. The micro-region parameter was considered when evaluating the existence of spatial aggregation. The temporal incidence of VL considering the patient's age was analyzed by linear regression using the following statistical model: *Z_t_* = *β*_0_ + *β*_1_*t* + *error* (*model 2*). The *Z*_*t*_ is the rate of cases per 100,000 inhabitants in year t, and β1 is the slope of the adjusted line that defined the secular trend of the incidence of VL for the subsequent year. A rising trend in cases/unit of time unit was observed when β1>0, whereas a downward trend in case rates/time was observed when β1<0. The case rate over time was stationary if β1 = 0. The model was adjusted in each of the following age groups: 0–4 years, 5–9 years, 10–19 years; 20–39 years and ≥40 years. The incidence/time unit was calculated within each age group.

### The impact of vaccination coverage on the incidence of VL

The impact of routine vaccination of the population, including vaccines for measles, poliomyelitis, BCG and hepatitis B, on the incidence of VL was analyzed in children under 5 years of age between 2000 and 2014. We evaluated these data for each micro-region. Vaccination rate data were superimposed on micro-region VL incidence data to determine whether vaccination coverage correlated with VL, particularly in children under age 5. We used an adjusted linear regression model as defined by the formula_:_ *Y_ti_* = *β*_0_ + *β*_1_*t* + *β*_2_*X_ti_* + *θ_i_MR*(*i*|19) + *error*, (*model* 3). Similar to *model 1*, the independent variable *Xti* was the vaccination coverage in micro-region *i* during year *t*. Because they were strongly correlated, an analysis of principal components was made and the vaccination coverage was represented by the score of the first component.

### The impact of nutritional status on the incidence of VL in children

The nutritional status was considered by the proportion *p*_*i*_ of children whose weight for age was categorized as very low(*p*_*1*_*)*, low*(p*_*2*_*)*, appropriate*(p*_*3*_*)* and high*(p*_*4*_*)*. From those, a Nutritional Status Index—NSI was built considering a weighted proportion with zero sum weights w = (-1, -1/3, 1/3, 1) defined by $NSI=-1p_{1}-(\frac{1}{3})p_{2}+(\frac{1}{3})p_{3}+1p_{4}$, ranging between -1 and 1. A value of NSI close to -1 point is a very low status while a value close to 1 point is a very high status. The influence of nutrition on VL development was assessed by a model similar to model 3, where *X*_*ti*_ is *NSI*_*ti*_. *(Model 4)*.

### Spatial relationships between canine and human VL, and between HIV and human VL

The relationship between the incidence of human VL with canine VL in micro-regions/time was performed by adjusting a simple linear regression model defined by the formula: *Y_t_* = *β*_0_ + *β*_1_*X_t_* + *error*, (*model* 5). The dependent variable, *Y*_*t*_ was set to log (rate human VL+ 0.5) in the year t in which there was canine examination, and the independent variable was *X*_*t*_ the corresponding level of canine infection (LCI). The level of canine infection was defined as a weighted proportion of infected dogs, using weights ranging from zero to 100 depending on the total number of dogs examined versus the number that had Leishmania infection, *model 6*, as follows:

$$LCI=log\left[(100\frac{infected}{examined})(100\frac{examined}{max(examined)})\right]$$

The spatial dependence and the association between response variables was performed by modeling the distribution of human VL case events with the spatial distribution of canine VL and social predictive factors, by adjusting mixed autoregressive spatial linear models (Spatial Lag Model Autoregressive-SAR) that captured the self-spatial correlation through a single ρ parameter (rho) added to the regression model; this was chosen to model factors in the same test: i.e., temporal variation in VL, effect of sex, and geographic micro-region. The equation was expressed as: *y* = *ρXy* + *XBeta* + *error*, (*Model* 7), where y is incidence of VL per 100,000 inhabitants at micro-region level. ρ (rho) measures the spatial dependence the VL incidence, W is the weight matrix modeling the spatial structure, X is the matrix of predictor variables, β is the regression coefficient vector which evaluated the association between Y and X, and *e*rror represents the residuals. The log transformation was applied to normalize the response distribution. The same analytical approach was applied to the spatial distribution of HIV/AIDS. The predictor variable data (X) was collected in the censuses 2000 and 2010. All statistical models tested herein are shown in S1 Supporting Information.

### Software used

We used Excel 2013 in the construction of the database, Statistica StatSoft version 7.0, in the estimation of linear models (Models 1 to 7) and Quantum GI version 2.12.e-Lyon (http://www.gnu.org.licenses) in the construction of maps and R System version 3.2.2 (https://www.r-project.org) for the mixed autoregressive linear models.

The source of base layers used to build the figures was found at http://censo2010.ibge.gov.br/resultados and https://mapas.ibge.gov.br/bases-e-referenciais/bases…/. The softwares used to build the maps were QGis version 2.12.e-Lyon (https://www.gnu.org/licenses/) and R System version 3.2.2 (https://www.r-project.org)

### Ethical considerations

This study was reviewed and approved by the Universidade Federal do Rio Grande do Norte Ethical review board CAAE12584513.1.0000.5537. Data were anonymous records and exempted from signed consent.

## Results

### Incidence and spatial distributions of human VL and AIDS in the state of Rio Grande do Norte, Brazil

**Fig 1:**
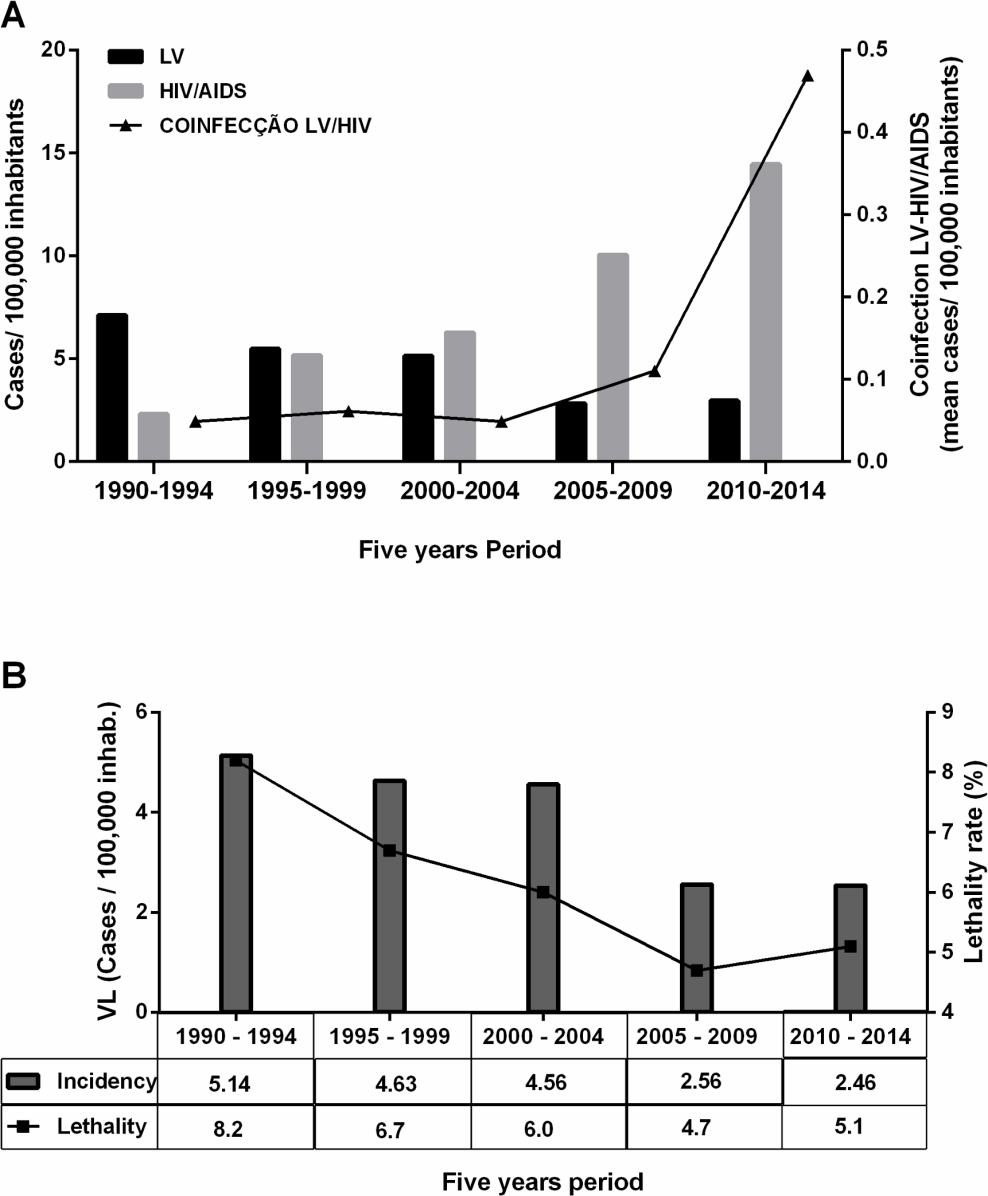
Analysis of a 25-year time series of visceral leishmaniasis, VL/AIDS and HIV in the state of Rio Grande do Norte, Brazil. **A.** Incidence of VL, AIDS, and VL-AIDS co-infections in Rio Grande do Norte 1990 to 2014. **B.** VL incidence by 100,000 inhabitants and the percent of VL lethality by five-year period.

A total of 3,252 cases of VL were reported in the state of Rio Grande do Norte, northeast Brazil, between the years of 1990 and 2014. The mean annual incidence was 4.6 VL cases/100,000 inhabitants. Fig 1A shows the incidence of VL, AIDS and VL/AIDS by 5-year period. The overall VL lethality rate was 6.4%, with a total of 210 deaths (210/3,252). However, the highest lethality rate was 8.2% in the period 1990–1994 and was associated with the highest incidence of VL (Fig 1B).

There were 5,777 cases of AIDS with an average incidence of 8.1 cases/100,000 inhabitants (Fig 1A) between 1990 and 2014. During the study period, the average incidence of concurrent VL/AIDS was 0.16 per 100,000 inhabitants. However, on the 5^th^ period (2010–2014) it reached 0.46/100,000 (Fig 1A).

**Fig 2:**
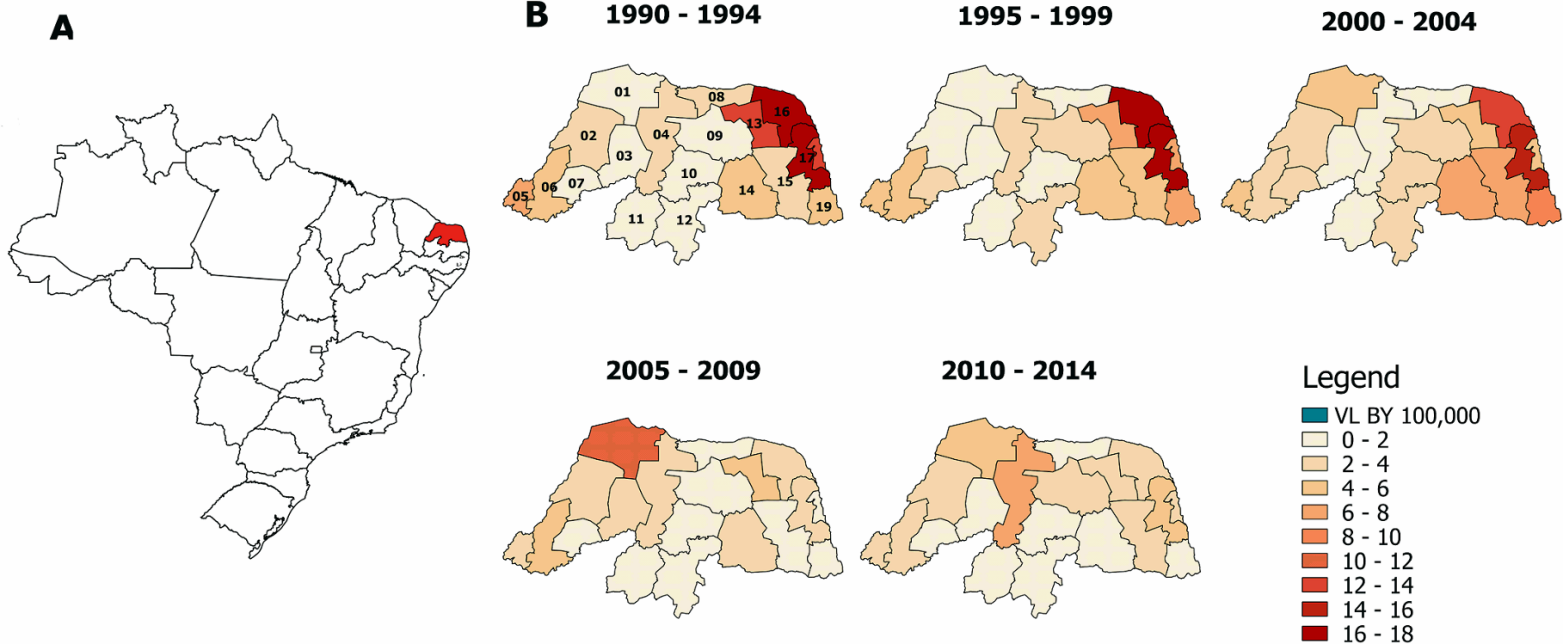
The spread of visceral leishmaniasis by micro region in a 25-year period. A. Map of Brazil showing in yellow the state of Rio Grande do Norte. Temporal and spatial distributions of human VL in the state of Rio Grande do Norte, 1990 to 2014, (cases/100,000 inhabitants).

VL cases were predominantly found between 1990–1994 in the eastern coastal region of Rio Grande do Norte, but the disease subsequently spread to the Northeastern Coast (θ_16_ = 5.983; p<0.0001) and to other areas (θ_17_ = 6.256; p<0.0001, *model 1)*, Table 1 and Fig 2. Although there has been an increase in areas reporting VL, there was a mean decrement of 0.135 VL cases/per year (β_1_ = -0.135, p<0.0001) (Table 1).

**Table 1:** Effect of time, sex and micro-region on the incidence of VL in accordance to *model 1*.

Predictors	Estimates of parameters β e θ	Standard Error	*P*	Density mean Inhab./Km^2^
Intercept (β_0_)	275.597	45.517	**<0.0001**	**-**
Year (β_1_)	**-0.135**	**.023**	**<0.0001**	**-**
[Sex = Male] (β_2_)	**2.498**	**.328**	**<0.0001**	**-**
Microrregions				
1- Mossoró (θ_1_)	0.427	1.011	0.673	340.8
2- Chapada do Apodi(θ_2_)	-2.405	1.011	**0.018**	85.4
3- MédioOeste(θ_3_)	-3.116	1.011	**0.002**	65.0
4- Vale do Açu(θ_4_)	-1.265	1.011	0.211	133.3
5- Serra de São Miguel(θ_5_)	0.260	1.011	0.797	302.0
6- Pau dos Ferros(θ_6_)	-1.289	1.011	0.202	208.2
7 -Umarizal(θ_7_)	-2.743	1.011	**0.007**	196.9
8- Macau(θ_8_)	-3.197	1.011	**0.002**	124.9
9- Angicos(θ_9_)	-1.927	1.011	0.057	61.7
10 -Serra de Santana(θ_10_)	-3.417	1.011	**0.001**	97.5
11 –Seridó Ocidental(θ_11_)	-3.761	1.011	**0.000**	146.7
12 -Seridó Oriental(θ_12_)	-2.840	1.011	**0.005**	151.4
13 -Baixa Verde(θ_13_)	1.608	1.011	0.112	149.1
14 –Borborema Potiguar(θ_14_)	-0.666	1.011	0.510	160.7
15- Agreste Potiguar(θ_15_)	-0.846	1.011	0.403	296.4
16—Northeastern littoral (θ_16_)	**5.983**	1.011	**0.000**	145.5
17—Macaíba(θ_17_)	**6.256**	1.011	**0.000**	586.4
18—Natal and perimetropolitan area (θ_18_)	0.950	1.011	0.347	10202.9

Model (1): *Y_ti_* = *β*_0_ + *β*_1_*t* + *β*_2_*I*(*Sex*) + *θ_i_MR*(*i*|19) + *error*

Reference: 19 –South coast (Density 415.5)

### Factors influencing human visceral leishmaniasis: Age, sex, nutrition, vaccination and canine visceral leishmaniasis

**Fig 3:**
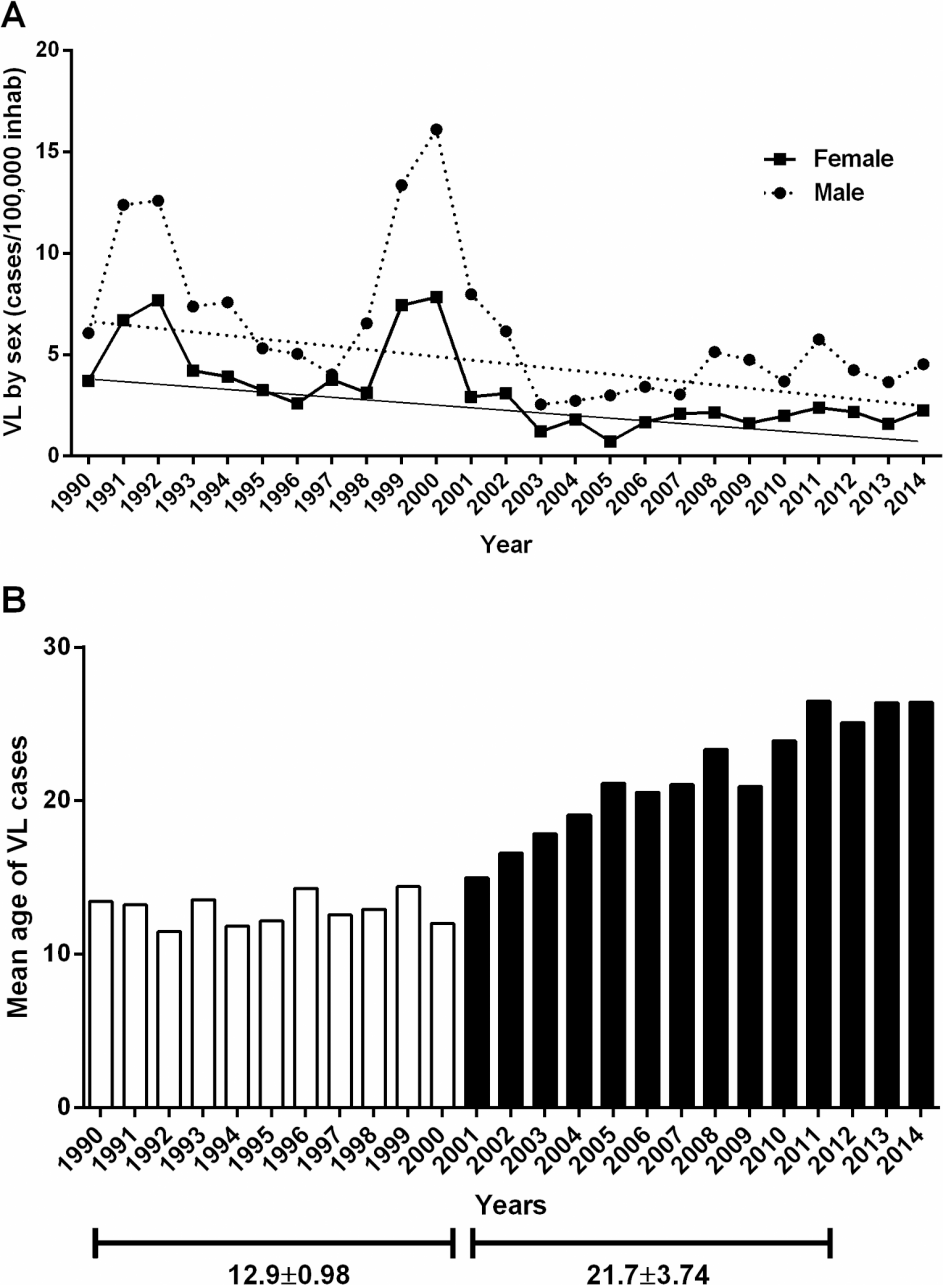
Incidence of VL by sex and year. **A.** The incidence was higher in males than females (Beta = 2.498, p<0.0001). **B.** Mean age of VL per year.

Over time there was a decreasing trend in the incidence of VL in both sexes. However, the male incidence was uniformly higher by approximately 2.5 per 100,000 (β2 = 2.498; p<0.0001), (Table 1; Fig 3A). There were two major peaks of VL, the first in 1991–1992 and the second in the 1999–2000 (Fig 3A).

The temporal incidence of VL decreased significantly 0–4 and 5–9 among the age groups (β_1_ = -0.0117, p<0.0001 and β_1_ = -0.0042, p<0.0001, respectively) between 1990 and 2014, with significant increase in the 20–39 and >40 age groups (β_1_ = 0.0071, p<0.0001 and β_1_ = 0.0105, p<0.0001, respectively), Table 2. At the same time, the VL incidence was stationary in the 10–19 age group (β_1_ = -0.0016, p = 0.1320), (Table 2, Fig 3B). The mean age of VL increased linearly during the period of study, (age = (-1392.657) + 0.704 (year), p<0.001), with an annual increase of 0.704 years (8.4 months). The mean age of VL prior to 2000 was 12.9 ± 0.98 (SD) years, whereas from 2000 to 2014, the mean age was 21.7 ± 3.74 (SD) years (p<0.005), Fig 3B.

**Table 2:** Mean temporal variation of VL cases / 100,000 inhabitants according to the age group^(^^*^^)^ (*model 2*).

Age group (years)	${\hat{\beta }}_{0}$(*intercept*)		${\hat{\beta }}_{1}(time\;coefficient)$		R^2^
	*Estimate*	p	*Estimate*	P	
0–4	0.5267	<0.0001	**-0.0117**	**<0.0001**	0.6564
5–9	0.1829	<0.0001	**-0.0042**	**0.0001**	0.4740
10–19	0.1470	<0.0001	-0.0016	0.1320	0.0958
20–39	0.1385	<0.0001	**0.0071**	**<0.0001**	0.5636
≥40	0.0049	0.7400	**0.0105**	**<0.0001**	0.8309

(*) Adjusting the model (2): *Z_t_* = *β*_0_ + *β*_1_*t* + *errorineachagegroup*

**Fig 4:**
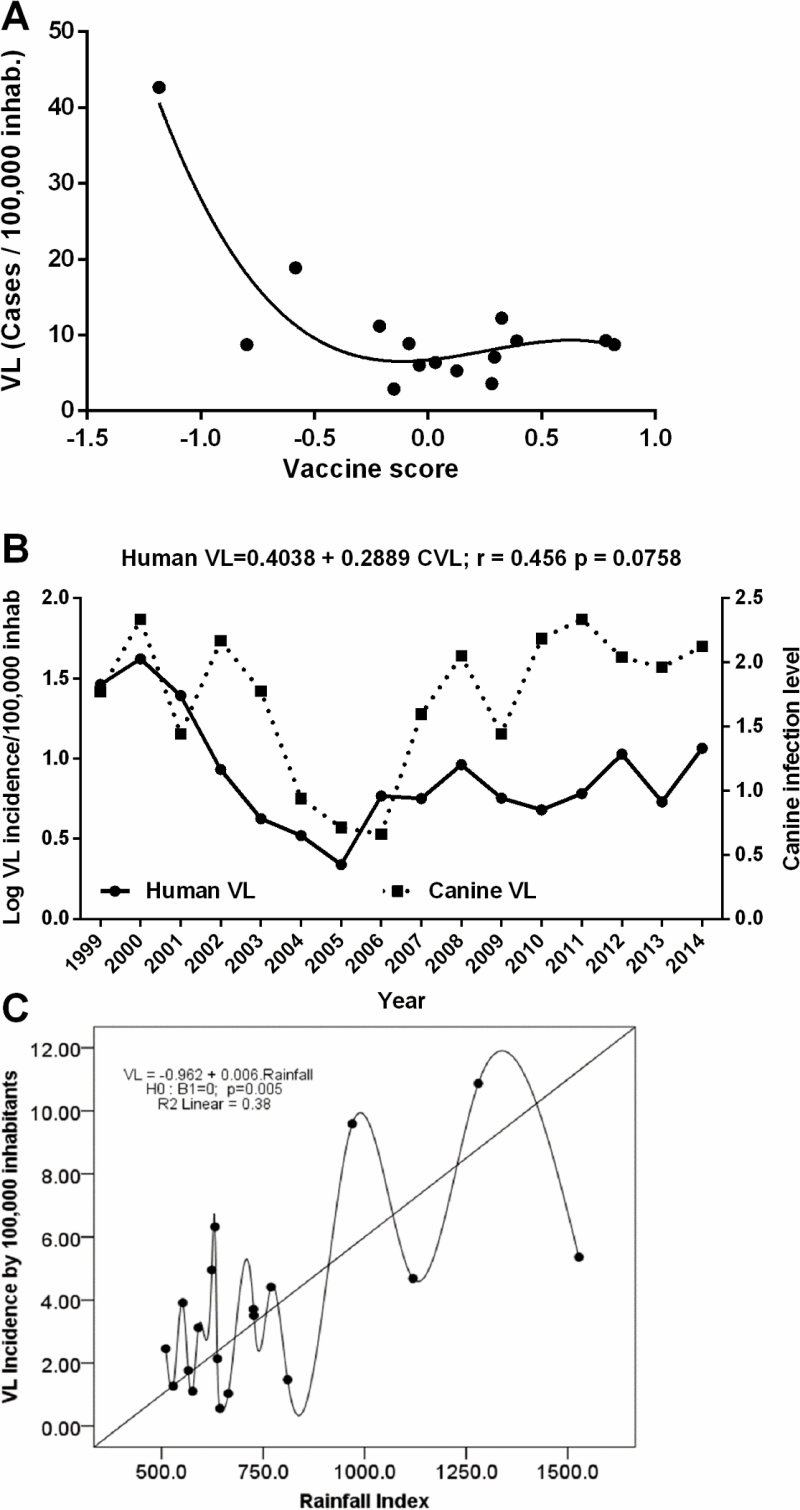
Risk factors for visceral leishmaniasis. **A.** Association of Vaccination coverage with decreased the incidence of visceral leishmaniasis in children. Association between VL incidence(y) and vaccine coverage (x). *y* = 6.574 + 11.481*x*^2^ − 10.13*x*^3^, *R*^2^ = 0.82. **B.** Correlation between human VL and *L*. *infantum* infected dogs. **C.** Variation in rainfall index and its relation to the incidence of VL.

The adjustment of *model 3* for spatial dispersion of VL showed that, by correcting for the effect of trends and differences between micro-regions, there was a strong negative association between the incidence of VL and the score of the vaccine coverage (β_2_ = -4.805; p = 0.0003). The score in year t and the micro-region i representing the vaccination coverage, at this time and place was calculated by X_ti_ = 0.0989*BCG_ti_* + 0.3993*POLIO_ti_* + 0.3181*MEASLES_ti_* + 0.3915*HEPATITIS_ti_*, obtained from the first principal component. This means that an increase in one unit in the vaccination coverage score was associated with a reduction of 4.8 in the incidence rate of VL in children younger than five years. There is a strong association between the two variables estimated by a third-degree polynomial relationship (Fig 4A).

The association between the Nutritional Status Index-NSI and the incidence rate of VL in children less than 5 years was evaluated by adjusting for other parameters, models 3 and 4. There was a trend toward an inverse correlation between better nutritional status and VL in children, but this did not reach statistical significance, (β_2_ = -141.76; p = 0.1136). In contrast, there was a positive linear correlation between the incidence of human VL and canine VL (Human VL *= 0*.*4038 + 0*.*2889LCI; r = 0*.*456*, *p = 0*.*0758)*, evaluated by the level of *L*. *infantum* canine infection (LCI) per models 5 and 6, as described in the methods session (Fig 4B). An increase of one LCI unit was associated with an increase in the incidence rate of human VL.

### Environmental determinants of visceral leishmaniasis

There was a positive correlation between the density of sand flies and the rainfall index (r = 0.762, *p*<0.0001), according to model 5. In addition, an increase in annual *rainfall index* correlated with increased annual incidence of VL (r = 0.616, *p* = 0.005). The fluctuation in rainfall index explained 38% of the variation in the incidence of VL, with a100 mm increment in annual rainfall associated with an increase of 0.6 in VL/100,000 inhabitants (Fig 4C).

### Socioeconomic determinants of human visceral leishmaniasis and HIV/AIDS

Among the socioeconomic variables that correlated with the incidence of VL was the percentage of households with garbage collection (β = -0.1684; p = 0.0116 in the 2000 census and β = -0.0341, p = 0.0153 in the 2010 census) and the percentage of households connected in the general water supply network (β = -0.3514; p = 0.100 in the 2010). In contrast, the incidence of AIDS correlated positively with garbage collection (β = 0.0358, p = 0.0005 in 2000 census and β = 0.0270, p = 0.0130 in the 2010 census), with higher sanitation level (β = 0.0407, p = 0.0007, in the 2000 census and β = 0.0418, p = 0.041in the 2010 census), with literacy rate (β = 0.0648, p = 0.0056 in 2010 census), with access to city water (β = 0.0431, p = <0.0001 in the 2000 census), adjusted *model 7*.

## Discussion

Transmission of VL occurs in settings where the infected sand fly vector lives in proximity to a mammalian reservoir and susceptible humans [36], or through other means of transmission such as blood transfusion [37]. Despite efforts of health officials to interrupt the routes of transmission of *L*. *infantum*, VL continues to be a major health problem in Brazil after Malaria [38]. The demographics have changed substantially since VL was first reported in the 1930’s [2]. During early years, the disease occurred predominantly in rural areas of the Northeast region, with most cases of VL occurring in children younger than 10 years [7]. Mass migration of the population to urban areas beginning in the 1980s was accompanied by a change in the pattern of transmission to peri-urban regions of large cities in the Northeast and the southeast regions of the country [39].

The state of Rio Grande do Norte in northeastern Brazil, provides an example of the changing epidemiology of VL. There was a significant increase in the age at disease diagnosis, with an increase in adult VL. The disease decreased in children under age 10 years and increased in adults, mainly from period 3 of this study. The average age at diagnosis of VL in Rio Grande do Norte rose from 12.9 years prior to 2000 to 21.7 years in 2014 (Fig 3B). An increase in the average age of VL has also been observed in other Brazilian states [39;40]. Human VL has been associated with poverty and malnutrition in children [20–22;41]. We hypothesized that multiple socioeconomic factors might contribute to the significant reduction in childhood VL in the less than 10 age groups. Since 1999, social programs to decrease poverty and economic measures to control inflation have been successfully implemented in Brazil, with a coincident improvement in many measures of health [42]. Interventions have included supplementation of micronutrients, including iron and vitamin A in pregnant women and children aged 6–18 months, as well as fortification of wheat and corn flours with iron and vitamins [43]. There is an increase in vaccine coverage, with more uniform administration of vaccines as polio, measles, BCG and others. Those measures have been associated with increased average birth weight [22;44], decreased childhood diarrheal diseases [45;46]. The improved health could lead to healthier gut brush border, better absorption of nutrients and protection against opportunistic pathogens such as *Leishmania*. As an example, studies have shown that the vaccine-preventable disease measles can induce immunosuppression for years [26;47].

In previous studies in the state of Rio Grande do Norte, we found that children and adults were infected at comparable rates with *L*. *infantum*, as detected by positive anti-leishmanial serology and/or positive skin test response to *Leishmania* antigens [15]. Since a majority of *L*. *infantum* infections are asymptomatic, it is likely that the above-mentioned health interventions have resulted in enhanced development of protective Type 1 immune responses to *Leishmania* spp. and other pathogens, with a decreased likelihood that *L*. *infantum* infection will progress to VL in young children. In addition, improved socioeconomic status, improved living conditions, and expansion of urban regions may be responsible for decreased sand fly density and transmission in some areas.

In the current study males accounted for most VL cases (67%). Greater susceptibility of males to VL has also observed in other human studies [8,40] and in experimental models of Leishmania infection, hamster and murine models of VL [48]. Higher levels of testosterone have been associated with increased risk of VL caused by *L*. *donovani* in India and Sudan [49;50] possibly mediated by increased IL-10 production and down regulation of Th1 responses.

Domestic dogs are thought to be the primary reservoir of *L*. *infantum* in Brazil [51]. A correlation between human and canine VL was observed in the current study, and in reports from other areas in Brazil and Northern Africa [52–54]. Further studies are needed to better define the roles of dogs and asymptomatically infected humans as reservoirs for *L*. *infantum* in the epidemiology of VL in Brazil.

Higher rainfall indices correlated temporally and geographically with a higher incidence of human VL, especially in areas close to the Atlantic Ocean. An association between VL with increased rainfall has been reported in other regions of Brazil [55;56]. However, some cases of VL occurred in areas with lower humidity, higher temperatures and lower rainfall indices. It is likely that variations in the microenvironment provided niches in which sand flies were nonetheless able to thrive in proximity to humans and a dog reservoir.

HIV/AIDS occurred predominantly in males in the initial stage of the pandemic, although recently more women have become infected with HIV in Brazil [57]. The highest incidence of HIV/AIDS occurs in urban regions, coinciding with regions that have a higher incidence of VL. HIV/AIDS has been expanding throughout Brazil since 1990, and has now spread to all areas of Rio Grande do Norte. Coinfection with HIV and *Leishmania* spp. has contributed to the increased incidence of VL in adults in southern Europe in Spain, France, Italy and Portugal [31;58;59]. Consistently, in this report we document the presence of VL/AIDS in Rio Grande do Norte since 1990, but there was a considerable increase in coinfection in the third period (2000–2004), presumably because HIV infections spread to areas that were endemic for *L*. *infantum* infection. A large number of individuals are asymptomatically infected with *L*. *infantum* in the state of Rio Grande do Norte [15;17;60], and people with asymptomatic *L*. *infantum* and HIV seem to be at greater risk of developing VL and of death [61]. Therefore, it is imperative that strategies and guidelines be developed to prevent the development of VL during HIV infection. In summary, the demographics of VL in northeastern Brazil have changed substantially over the past 25 years. The incidence has decreased in children in association with improved nutrition, socioeconomic status, childhood immunizations, and overall health. In contrast, the incidence of VL in adults has increased. The latter could be explained in part by failure to develop immunity to the parasite as a child, and the geographic coincidence of HIV infection and VL. The emergence of concurrent VL-AIDS poses a serious health challenge for the future.

## Supporting information

S1 Supporting Information Descriptions of the models used in this study. (DOCX)

S2 Supporting Information STROBE checklist. (DOC)
