# Supplementary material for: Changing demographics of visceral leishmaniasis in northeast Brazil: Lessons for the future
Source: PLoS Negl Trop Dis. 2018 Mar 6;12(3):e0006164. doi: 10.1371/journal.pntd.0006164 (PMC5839541; doi:10.1371/journal.pntd.0006164)
Supplement: S1 Supporting Information — (DOCX) [file pntd.0006164.s001.docx]

Supporting Information 1.Descriptions of the models used in this study.

Model 1 . $Y_{ti}=\beta_{0}+\beta_{1}t+\beta_{2}I\left( Sex \right)+ \theta_{i}MR\left( i | 19 \right)+ error$

Model 2. $Z_{t}= \beta_{0}+\beta_{1}t+error$

Model 3. $Y_{ti}=\beta_{0}+\beta_{1}t+\beta_{2}X_{ti}+\theta_{i}MR\left( i | 19 \right)+error$

Model 4. w=(-1, -1/3, 1/3, 1) defined by $NSI=-1p_{1}-(\frac{1}{3})p_{2}+(\frac{1}{3})p_{3}+1p_{4}$

Model 5 $Y_{t}=\beta_{0}+\beta_{1}X_{t}+ error$.

Model 6. $\boldsymbol{LCI=log}\left[ \left( \boldsymbol{100}\frac{\boldsymbol{infected}}{\boldsymbol{examined}} \right)\left( \boldsymbol{100}\frac{\boldsymbol{examined}}{\boldsymbol{max(examined)}} \right) \right]$

Model 7. $y=\rho Xy+XBeta+error$
